# Galectin-8 deficiency promotes chronic splenomegaly persistence in Chagas disease

**DOI:** 10.3389/fcimb.2025.1625938

**Published:** 2025-10-01

**Authors:** Adriano Bertelli, Juan Ignacio Saborit, Cristian Gabriel Beccaria, Laura Vanagas, Sergio Oscar Angel, Oscar Campetella, Adriana Gruppi, María Susana Leguizamón

**Affiliations:** ^1^ Instituto de Investigaciones Biotecnológicas (IIBio), Consejo Nacional de Investigaciones Científicas y Técnicas (CONICET), Escuela de Bio y Nanotecnologías (EByN), Universidad Nacional de San Martín (UNSAM), Buenos Aires, Argentina; ^2^ Centro de Investigaciones en Bioquímica Clínica e Inmunología (CIBICI), Consejo Nacional de Investigaciones Científicas y Técnicas (CONICET), Córdoba, Argentina; ^3^ Instituto de Investigaciones Tecnológicas de Chascomús (INTECH), Consejo Nacional de Investigaciones Científicas y Técnicas (CONICET), Escuela de Bio y Nanotecnologías (EByN), Universidad Nacional de San Martín (UNSAM), Buenos Aires, Argentina

**Keywords:** Chagas disease, Galectin-8, inflammation, splenomegaly, Trypanosoma cruzi

## Abstract

Galectins (Gals) are mammalian lectins with affinity for *β*-galactosides, which drive the immune response through several mechanisms. The specific role of Gal-8 in the development of inflammation remains controversial, as it has been shown to induce either T cell proliferation or regulation in different models. During the acute phase of *Trypanosoma cruzi* infection, a characteristic splenomegaly is induced that is associated with both antigen-specific and non-specific polyclonal lymphocyte proliferation. This splenomegaly resolves as the infection transitions to the chronic phase. While the pathogenesis of Chagas disease is not yet fully understood, it is widely accepted to involve both parasite persistence and the host immune response. In this study, C57BL/6J and Gal-8-deficient (KO) mice infected with the Ac strain were analyzed during the chronic phase (4 months post-infection). Notably, infected Gal-8KO mice failed to resolve the *T. cruzi*-induced acute phase splenomegaly. Despite this, parasitemia, spleen parasite load, and survival rates were comparable between the two groups, suggesting that Gal-8 is not involved in parasite control. The observed differences in spleen cellularity were primarily attributed to T lymphocyte proliferation, while B cells exhibited no significative changes in total cell number, proliferation levels and production of total and parasite-specific antibodies. Overall, our results reveal that Gal-8 plays an anti-inflammatory role during chronic *T. cruzi* infection and is critical in controlling splenomegaly, a process for which no associated regulatory molecules have been identified to date.

## 1 Introduction

*Trypanosoma cruzi* is an intracellular protozoan parasite, the etiologic agent of Chagas disease, that affect 7 million people worldwide. It is a main public health problem in Latin America, which spreads to developing countries, mainly through blood transfusion from infected patients who migrates to non-endemic areas (de Sousa et al., 2024). The parasite induces the development of chronic megaviscera and/or cardiomyopathy in ∼30% of patients. Chronic Chagas cardiomyopathy is the most frequent and severe, and is a consequence of an intense, and permanent inflammation process throughout the infection caused by local parasite persistence (Garcia et al., 2005; Marin-Neto et al., 2007). It has been accepted that the parasitic load is responsible for triggering the inflammatory response (Tarleton and Zhang, 1999).

Galectins (Gals) are mammalian lectins containing conserved carbohydrate recognition domains (CRDs) that interact with ß-galactose-containing glycans displayed on different cells. Galectin-8 (Gal-8) together with Gal-4 and Gal-9, belong to the tandem-repeated group. Gal-8 contains two CRDs, with different glycan specificity, that are fused via a peptide linker (Bidon et al., 2001) and, unlike other Gals, is widely distributed in different tissues under normal conditions, as well as in tumors and synovial fluid (Tribulatti et al., 2020). It is also expressed in endothelium and released into the environment when tissue is damaged (Thijssen et al., 2008; Cattaneo et al., 2014) and has been involved in different events, both pathological and homeostatic (Elola et al., 2014). It has been communicated Gal-8 ability to bind to surface ligands of *T. cruzi* (Pineda et al., 2015) as well as to exposed glycans in damaged bacterial-infected vacuoles, acting as danger receptors (Thurston et al., 2012). Gal-8 plays both pro- or anti-inflammatory roles depending on the context (Reviewed in (Tribulatti et al., 2020), see also (Zick, 2022). We have reported the association of Gal-8 in inducing T cell expansion under both antigen-specific and nonspecific conditions and showed that this T cell co-stimulation is dependent on Gal-8 concentration (Tribulatti et al., 2009). On the other hand, an anti-inflammatory effect has been attributed to Gal-8 in autoimmune diseases such as rheumatoid arthritis (Eshkar Sebban et al., 2007), as well as in uveitis and encephalomyelitis models (Sampson et al., 2015; Pardo et al., 2017). In line with this, we demonstrated the anti-inflammatory role of Gal-8 during chronic *T. cruzi* infection by examining Gal-8-deficient (Gal-8KO) mice infected with the *T. cruzi* Ac strain (4 months post-infection, mpi) (Bertelli et al., 2020). While survival and parasitemia rates were similar between Gal-8KO-infected mice and wild-type (WT) C57BL/6J control mice, the absence of Gal-8 resulted in significant inflammation in the heart, skeletal muscle and liver, independently of parasite load. Remarkably, we observed an increased presence of neutrophils and macrophages in cardiac tissue of infected Gal-8KO (iGal-8KO) mice. The latter is associated with the absence of Gal-8-dependent preaparesis. These findings highlight the anti-inflammatory role that Gal-8 exerts during chronic *T. cruzi* infection (Bertelli et al., 2020).

During the early stages of infection, *T. cruzi* employs sophisticated strategies to evade and modulate the host immune response. Although unable to eliminate the parasite, the specific immune response allows the host survival and development of chronicity. Th1 effector mechanisms are relevant to parasite control (Kumar and Tarleton, 2001; Hoft and Eickhoff, 2002; Volta et al., 2021), but orchestrating a balanced Th1 and Th2 immune response is crucial in *T. cruzi* infection, since an excessive or dysregulated activation, can lead to host tissue damage (Ruiz Diaz et al., 2015; Llaguno et al., 2019). The role of Th17 in protection, in murine and human studies have been also communicated (Magalhaes et al., 2013; Cai et al., 2021; Duarte et al., 2025). CD8^+^ T cells play a central role in controlling the infection by targeting and eliminating the infected cells (Araujo Furlan et al., 2018). However, in the chronic phase, *T. cruzi* modulates the effectiveness of CD8^+^ T cell responses, leading to incomplete pathogen clearance and immune exhaustion (Albareda et al., 2013). Regulatory T (Treg) cells play a dual role by helping to suppress excessive immune activation that could damage host tissue, while also aiding tissue repair once the infection is controlled. A transient decrease in Treg cells, during the acute phase, allows for a stronger activation of CD8^+^ T cell responses, which is crucial for controlling the parasite (Araujo Furlan et al., 2018). B cell response and antibodies are important in targeting and controlling circulating parasites (Umekita and Mota, 2000; Gorosito Serran et al., 2017).

A prominent feature of acute *T. cruzi* infection is splenomegaly (Lopes et al., 1995; Cabrera et al., 2013; Dehesa-Rodriguez et al., 2022), driven by intense polyclonal proliferation of B cells, CD4^+^ and CD8^+^ T cells. This hyperactivation, which includes both antigen-specific and non-specific responses, contributes to immune dysregulation and facilitates parasite dissemination (Minoprio, 2001). Although the triggers for this massive lymphocyte expansion are not fully understood, parasite-derived components have been associated (Reina-San-Martin et al., 2000; Montes et al., 2002; Montes et al., 2006). Interestingly, splenomegaly typically resolves during the transition from acute to chronic infection, with the spleen regaining its normal size and function. Our study identifies Gal-8 as a critical regulator in this process. The absence of Gal-8 results in persistent splenomegaly during chronic *T. cruzi* infection. Given the importance of lymphocyte hyperactivation, often described as a polyclonal response during the acute phase, we conducted an in-depth analysis of the cellular events underlying chronic splenomegaly. Our findings highlight Gal-8 as a pivotal player in controlling splenic size and immune homeostasis.

## 2 Materials and methods

### 2.1 Ethics statement

The study adhered to the principles of the Basel Declaration. Protocols No. 10/2017 and 08/2022 were approved by the Committee for Experimental Animal Care and Use (CICUAE) of the Universidad Nacional de San Martín (UNSAM), following the recommendations of the *Guide for the Care and Use of Laboratory Animals* of the National Institutes of Health (NIH).

### 2.2 Mice

Male C57BL/6J (B6) mice were sourced from our in-house colony, established using breeder pairs obtained from The Jackson Laboratory (Bar Harbor, ME, USA). Male mice deficient in Gal-8 *Lgals8* gene [B6; 129S5-*Lgals8Gt* (OST314218) Lex/Mmucd] were acquired as heterozygotes from the Mutant Mouse Resource & Research Centers (MMRRC; University of California, Davis, CA, USA). After 12 in-house backcrosses to B6, a homozygote Gal-8 knock-out (Gal-8KO) colony with >95% of B6 genetic background was established, as assessed by The Jackson Laboratory Genotyping Resources. CF1 mice were bred from a colony obtained from Charles River Company. Mice were anesthetized with isoflurane, before manipulation. At least 4–5 animals were included in each group.

### 2.3 *Trypanosoma cruzi* parasites and experimental infection

Male mice 10 to 16 weeks old were intraperitoneally infected with 50,000 *T. cruzi* Ac strain blood-derived trypomastigotes (DTU TcI) (Risso et al., 2004); which is maintained through serial passages in CF1 mice. Parasitemia was evaluated by counting trypomastigotes using a hemocytometer. Analysis of Gal-8 roles in *T. cruzi* murine model were conducted at 4 months post-infection (mpi). Age-matched wild type B6 (WT), and Gal-8KO mice were included as non-infected controls.

### 2.4 *Toxoplasma gondii* parasites and experimental infection

Male mice aged 10 to 16 weeks were orally infected with 5 *T. gondii* Me49 strain cysts (Fux et al., 2003); which are maintained through serial passages in mice. Parasite load was evaluated by counting cysts in brain samples. Brains were homogenized in 2 ml of PBS with a Dounce tissue grinder. The number of cysts per brain was counted under an optical microscope in 3 x 20 µl aliquots of each homogenized brain. Mice were analyzed at 45, 60, 90 days post-infection (dpi) (de Medeiros Brito et al., 2022). Age-matched WT mice and Gal-8KO mice served as non-infected controls.

### 2.5 Flow cytometry

Spleens were mechanically disaggregated using a mesh, cells were centrifuged and incubated in red cells lysis buffer (Sigma) for 5 min at room temperature (RT). Cells were resuspended in PBS 2% FBS. Splenocytes were counted after erythrocyte lysis. Viable cell numbers were determined by trypan blue dye exclusion. We determined the total splenocyte number using a Neubauer chamber and expressed as total cells/spleen and as cells/ml. The percentage of each cell subpopulation was established by flow cytometry and subsequently the total number of cells in each subpopulation per spleen following the formula: % of cell subpopulation obtained by cytometry x total number of splenocytes (obtained by Neubauer chamber counting)/100 = total number of cell subpopulation in the spleen (Dual-platform method) (Cossarizza et al., 2021). For surface staining, cell suspensions were incubated with fluorochrome-labeled Abs (BioLegend) along with Live/Dead Fixable Aqua 405 (Invitrogen, dilution 1/400) in ice-cold PBS 2% FBS for 45 minutes at 4˚C. Samples were then washed, resuspended, and 50,000 events were acquired by flow cytometry. For intracellular staining, after surface staining, cells were fixed and permeabilized according to the manufacturer’s instructions using the Foxp3/Transcription Factor Staining Buffer Set commercial kit (eBioscience). Subsequently, samples were incubated for 30 min at RT with specific antibodies for transcription factors. Finally, cells were washed, resuspended and 50,000 events were acquired by flow cytometry. After doublet exclusion, lymphocytes were identified based on forward (FSC-A) and side scatter (SSC-A) parameters. Live cells were gated by excluding those stained with Live/Dead Fixable Aqua 405. The following antibodies were used: FITC-labelled anti-mouse CD3, CD11c, CD21, CD44 and CD19; PE-labelled anti-mouse CD19, B220, Fas, CD11b, CTLA-4, Ki-67, and F4/80; PerCP-Cy5.5-labelled anti-mouse CD11b, CD23, CD138, CD62L, MHCII, F4/80 and Foxp3; APC-labelled anti-mouse CD8, GL7, Ly6C, Ly6G, CXCR5, and CD39; PE-Cy7-labelled anti-mouse CD19, CD206, B220, Ly6G, ICOS, and CD8; and APC-Cy7-labelled anti-mouse CD4, CD11c, Ly6C, and B220 (all from BioLegend). Samples were acquired using FACS Canto II and Fortessa cytometers (Becton Dickinson) and data were analyzed using FlowJo V10 software (BD).

### 2.6 Proliferation assays

Splenocytes, 5x10^5^ cells, were seeded in 96-well plates with RPMI 1640 plus 10% FBS, with or without *T. cruzi*-specific antigens stimulation (1µg). *T. cruzi* trypomastigote pellets underwent 5 successive rounds of freezing and thawing using liquid nitrogen. Eighteen hours before harvesting, 50 µL of complete RPMI 1640 medium containing 1µCi of [*methyl*-^3^H]-thymidine (New England Nuclear) was added/well. Cells were harvested using a semi-automatic cell harvester (Inotech Bioscience). Incorporated radioactivity was analyzed by recording cpm for each well. All treatments were performed in quadruplicate, and data were expressed as mean ± SEM.

### 2.7 Parasite load quantification

Genomic DNA was purified from spleens using DNAzol (GIBCO) reagent following the manufacturer’s instructions. *T. cruzi*-specific DNA primers: TCZ-Forward 5’-GCTCTTGCCCACAMGGGTGC-3’ (where M=A/C) and TCZ-Reverse 5’-CCAAGCAGCGGATAGTTCAGG-3’, which amplify a 182bp product, were used to quantify parasitic load by real-time qPCR employing SYBR Green (Applied Biosystems). Simultaneously, reactions containing 50 ng of mouse genomic DNA and 0.5 µM of murine tumor necrosis factor (TNF) primers: TNF-5241 5’-TCCCTCTCATCAGTTCTATGGCCCA-3’ and TNF-5411 5’-CAGCAAGCATCTATGCACTTAGACCCC-3’ were used as loading controls. Primer sequences were previously described by Cummings and Tarleton (2003). Results were expressed as parasite equivalents/50ng DNA.

### 2.8 ELISA

Splenocyte culture supernatants were collected, and cytokine secretion of IL-2, IL-4, IL-6, IL-10, IL-17, TNF and IFNγ was measured by ELISA following the manufacturer’s instructions (BioLegend) and using recombinant cytokines standard curves. Briefly, 96-well plates were coated with the corresponding antibody in phosphate buffer (pH 9) overnight (ON) at 4 °C. Blocking was performed using TBS-Tween 20 (0.05%) with BSA (5%) for 1h at 37 °C. Samples dilutions were then incubated for 2h at RT. Detection biotinylated antibodies were incubated in TBS-BSA (0.1%) for 1h at RT. Streptavidin-HRP conjugate (BioLegend) diluted 1:5000 was added for 1h at RT in the dark. Washing steps were performed with TBS-Tween 20 (0.05%) three times. Colorimetric reaction was developed with TMB (Sigma) and hydrogen peroxide in 10 mM citrate buffer (pH 5.5). Finally, the reaction was halted with 0.2 M sulfuric acid, and absorbance at 450 nm was measured using a FilterMax F5 (Molecular Devices).

For total IgG assays, plates were first coated with goat anti-total mouse immunoglobulins from Sigma. Seven serial dilutions (from 1:40 to 1:2560) of each serum sample were incubated for 2h. Washing steps were performed with TBS-Tween 20 (0.05%) three times. Anti-mouse IgG HRP-conjugated antibodies (BioLegend) were then added and incubated for 1h at RT. Colorimetric reaction was developed and measured as above. The cut off was determined using a serum pool obtained from uninfected mice from both groups. Avidity of sera taken from infected mice (4mpi) was assayed on *T. cruzi-*antigens*-*sensibilized plates (Wiener labs, Argentina) (Marcipar et al., 2001). Sera were tested at 1/5,000 dilution, that rendered OD_450_ about 0.5, and after washing, a 6M urea in PBS solution was added at room temperature for 15 min. After washings, anti-mouse IgG HRP-conjugated antibodies (BioLegend) were then added at 1/5,000 dilution incubated for 1h and colorimetric reaction was developed and measured as above. The values are expressed as the ratio between the absorbance obtained without and with treatment with 6M urea. To determine IgG subclasses, samples were incubated for 2h on *T. cruzi-*antigens*-*sensibilized plates (Wiener labs, Argentina) and, after washings, biotinylated antibodies specific for the different subclasses (BioLegend) were added and then revealed with HRP-conjugated streptavidin (BioLegend). Colorimetric reaction was developed and measured as above.

### 2.9 Histochemical assays

Spleens from infected and uninfected mice were frozen in liquid nitrogen. Seven μm thick cryosections were obtained and then fixed in acetone for 10 min at -20 °C and air-dried for 10 min. They were then rehydrated in TRIS buffer and blocked for 30 min at RT with TRIS-10% normal mouse serum. Subsequently, sections were incubated for 1h at RT in a humid chamber with different combinations of fluorochrome-conjugated antibodies in TRIS buffer. Finally, sections were mounted with FluorSave (Merck Millipore) and observed under an Olympus FV 1000 confocal microscope. Images were processed with Adobe Photoshop.

Antibodies and reagents used were Alexa Fluor 488-labelled anti-mouse CD4 and Alexa Fluor 647 labelled peanut agglutinin (PNA, Invitrogen), and PE-labelled anti-mouse B220 (eBioscience).

### 2.10 Quantitative analysis of GC architecture

This analysis was performed using *QuPath* software (version 0.5.1). For each spleen section, three representative fields containing clearly identifiable B cell follicles (B220^+^) were selected. B cell follicles were manually annotated based on B220 staining, while GCs were identified and annotated based on strong PNA positivity and concurrent downregulation of B220 expression. Regions of interest (ROIs) corresponding to follicles (yellow) and GCs (cyan) were defined manually for all groups in a blinded fashion. The following parameters were measured: Follicle area (µm²): total area of each annotated B220^+^ follicle; GC area (µm²): total area of PNA^+^ regions within each follicle; GC area per field (µm²): total GC area normalized to the entire field of view; GC/follicle ratio: calculated as the ratio of total GC area to its corresponding follicle area for each ROI pair; GC circularity: used as a geometric metric of structural organization, calculated using the formula: Circularity = (4 × π × Area)/(Perimeter²). Circularity values range from 0 to 1, with values closer to 1 indicating more regular, circular shapes. Circularity was computed in QuPath via a custom Groovy script that extracted area and perimeter measurements and stored circularity as a new annotation measurement. Data from at least three independent animals per group were pooled for quantification. Measurements were exported from QuPath and analyzed in GraphPad Prism for statistical analysis.

### 2.11 Statistics

Statistical significance of comparisons of mean values was assessed by Shapiro-Wilk test for normality then by using two-tailed Student’s *t*-test and ANOVA, followed by Bonferroni’s *post-hoc* test and a Gehan-Breslow-Wilcoxon test. Data in follicles area were analyzed using one-way ANOVA with Šídák’s multiple comparisons test. All assays were performed with GraphPad Prism software. At least 4–5 animals were included in each group.

## 3 Results

### 3.1 The absence of Gal-8 enables the persistence of chronic splenomegaly during *T. cruzi* infection

**Figure 1:**
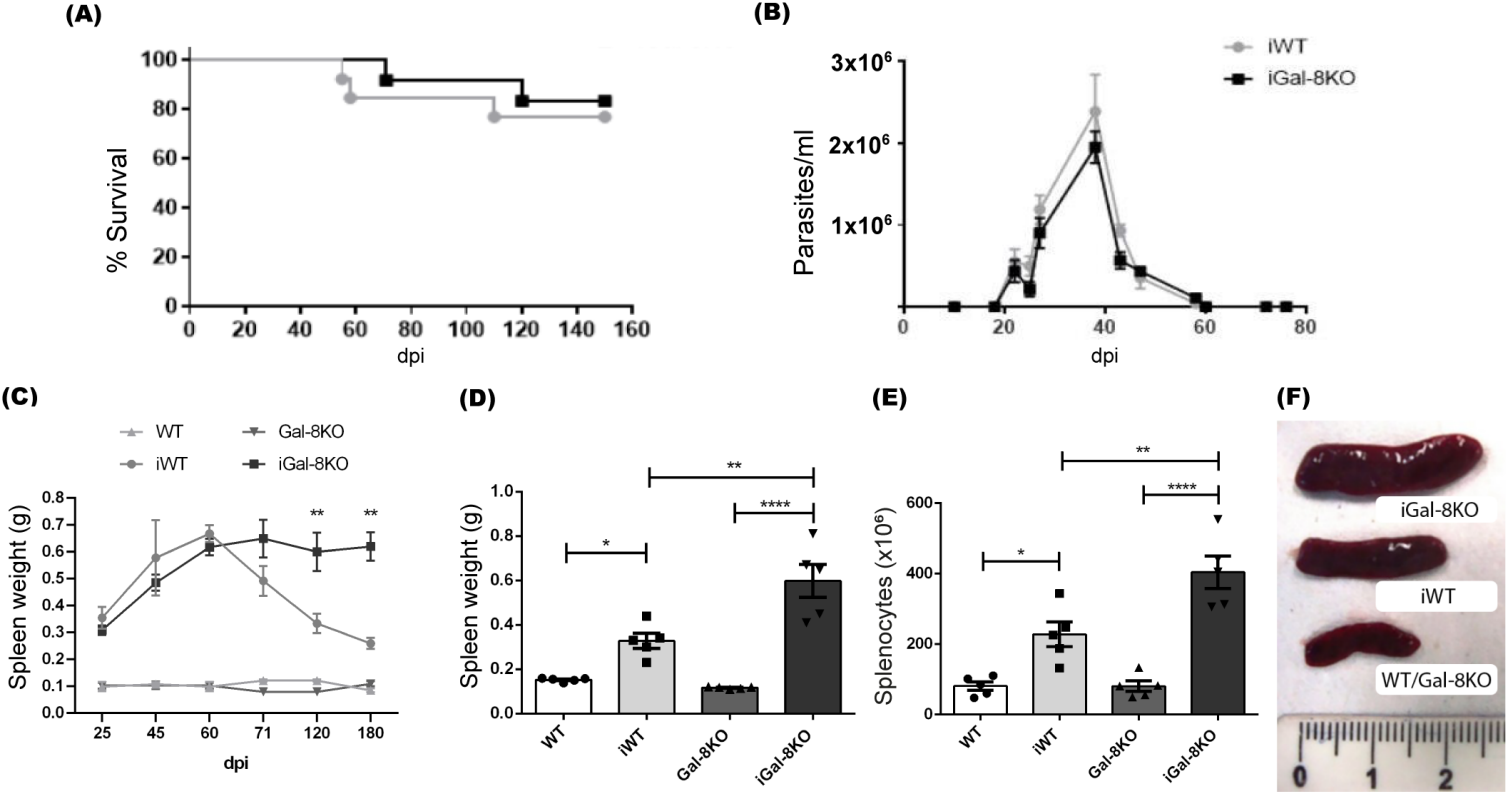
iGal-8KO mice showed persistent splenomegaly in the chronic phase of infection. WT and Gal-8KO mice were infected with Ac strain of T. cruzi. **(A)** Survival curves. **(B)** Parasitemia curves. **(C)** Weight of spleens obtained along the infection and of uninfected mice. D-F obtained at 4 mpi: **(D)** Spleen weight, **(E)** Total number of cells/spleen. **(F)** Macroscopical image of spleens from infected mice. Spleens of uninfected controls are shown as reference. No variations in body weight or parasite load were found between infected WT or KO mice. Rule is in cm. Data are expressed as mean ± SEM of a representative assay. At least three independent experiments were performed. Statistical significance of comparisons of mean values was assessed using one-way ANOVA followed by Bonferroni’s post-hoc test. *p<0.05; **p<0.01; ****p<0.0001.

To investigate the role of Galectin-8 (Gal-8) in the inflammatory environment induced by *T. cruzi*, we used a chronic infection model. Gal-8-deficient (Gal-8KO) and wild-type (WT) mice were infected with *T. cruzi* Ac strain, model in which we previously observed comparable parasitemia levels and survival rates (80%) between the two experimental groups (**Figures 1A, B**) (Bertelli et al., 2020). The development of cardiac inflammation and fibrosis was observed, as expected for *T. cruzi* chronic infection (at 4 mpi), although the absence of Gal-8 enhanced the inflammation level (Bertelli et al., 2020).

According to previous reports indicating that *T. cruzi* induces splenomegaly (Lopes et al., 1995; Dehesa-Rodriguez et al., 2022), both iWT and iGal-8KO mice exhibited pronounced splenomegaly during the acute phase of the infection. In agreement with other murine models **((** de Alba Alvarado et al., 2023) and reviewed in Talvani and Teixeira (Talvani and Teixeira, 2011)**),** in our model the acute phase last at 2 mpi when parasitemia values became undetectable by microscopic analysis and the splenomegaly resolution starts in the iWT mouse (**Figures 1B, C**). However, spleens of iGal-8KO mice showed significant increase that persisted as long as 6 mpi when compared to those from iWT mice (**Figure 1C**). Measures of weight, total number of splenocytes together with an illustration of the spleens collected at 4 mpi are shown in **Figures 1D-F**. This unexpected result suggests that the absence of Gal-8 disrupts the resolution of spleen expansion during the chronic phase of infection. All the assays of this study were conducted at 4mpi.

It is noteworthy that body weight of iWT and iGal-8KO mice remained similar throughout the infection. The parasite load, assessed by qPCR, was also similar between both groups (iWT 1,9 ± 0,9 *vs*. iGal8KO 2,3 ± 0,9 parasite equivalents/50ng DNA). Furthermore, spleens weight from non-infected Gal-8KO mice exhibited similar values to those from non-infected WT mice.

### 3.2 Cellular immune populations implicated in chronic splenomegaly

To identify the cellular populations contributing to the persistence of chronic splenomegaly observed in iGal-8KO mice, we conducted an in-depth analysis of immune splenic cells at 4 mpi, a time point when splenomegaly is being resolved in iWT mice.

#### 3.2.1 Myeloid cells number is increased in the spleen of iGal-8KO mice

**Figure 2:**
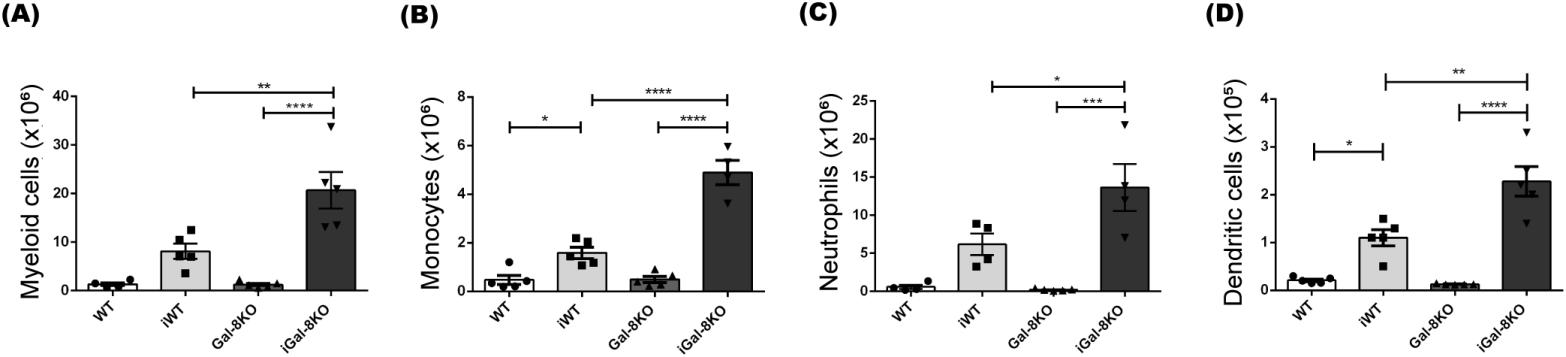
iGal-8KO mice showed increased number of myeloid cells. WT and Gal-8KO mice were infected with Ac strain of *T. cruzi.* At 4 mpi cells were obtained from spleens of iWT and iGal-8KO mice and their respective uninfected control groups. Statistical analysis of absolute number of: **(A)** Myeloid cells/spleen (CD11b^+^), **(B)** Monocytes/spleen (CD11b^+^ Ly6C^+^ Ly6G^-^), **(C)** Neutrophiles/spleen (CD11b^+^ Ly6C^+^ Ly6G^+^) and **(D)** Dendritic cells/spleen (CD11c^+^ F4/80^-^). Data are expressed as mean ± SEM of a representative assay. At least three independent experiments were performed. Panel used for flow cytometry staining: CD11c-FITC; CD11b-PE; F480-PerCP; Ly6G-PE-Cy7; and Ly6C-APC. Statistical significance of comparisons of mean values was assessed using one-way ANOVA followed by Bonferroni’s *post-hoc* test. *p<0.05; **p<0.01; ***p<0.001; ****p<0.0001.

The analysis of splenic myeloid cells, characterized by the CD11b surface marker, showed an increase in absolute numbers in iGal-8KO mice compared to iWT, with no differences observed between uninfected groups (**Figure 2A**). Further analysis of myeloid subpopulations revealed that the spleens of iGal-8KO mice exhibited significantly higher absolute numbers of monocytes (CD11b^+^ Ly6C^+^ Ly6G^-^), neutrophils (CD11b^+^ Ly6G^+^ Ly6C^+^), and dendritic cells (CD11c^+^ F4/80^-^) compared to the spleens of iWT mice (**Figures 2B-D**). In contrast, the absolute number of macrophages (CD11b^+^ F4/80^+^) was similarly increased in both iWT and iGal-8KO groups (iWT: 3.4± 0.7; iG8: 4.9 ± 0.65) while no differences were observed between uninfected mice (WT: 0.5 ± 0.14; G8: 0.6 ± 0.11).

#### 3.2.2 Impact of Galectin-8 deficiency on B cell function and germinal center formation

Considering the immunomodulatory role that many proteins from the galectin family play in the B cell immune response (Sharma et al., 2011; Tsai et al., 2011; Rabinovich and Croci, 2012; Beccaria et al., 2018), and given that the predominant leukocyte population in this organ is composed of B cells, we conducted a detailed analysis of this population. Examination of the B compartment revealed that, although infected mice showed higher absolute numbers of B cells compared to their uninfected controls, there were no differences between iWT and iGal-8KO mice (**Supplementary Figure 1**). Since the B compartment is composed of different subsets that fulfill diverse functions (LeBien and Tedder, 2008), we next assessed the representation of each subpopulation in the spleen of infected and uninfected mice using the B220, CD19, CD21 and CD23 antibody panel.

**Figure 3:**
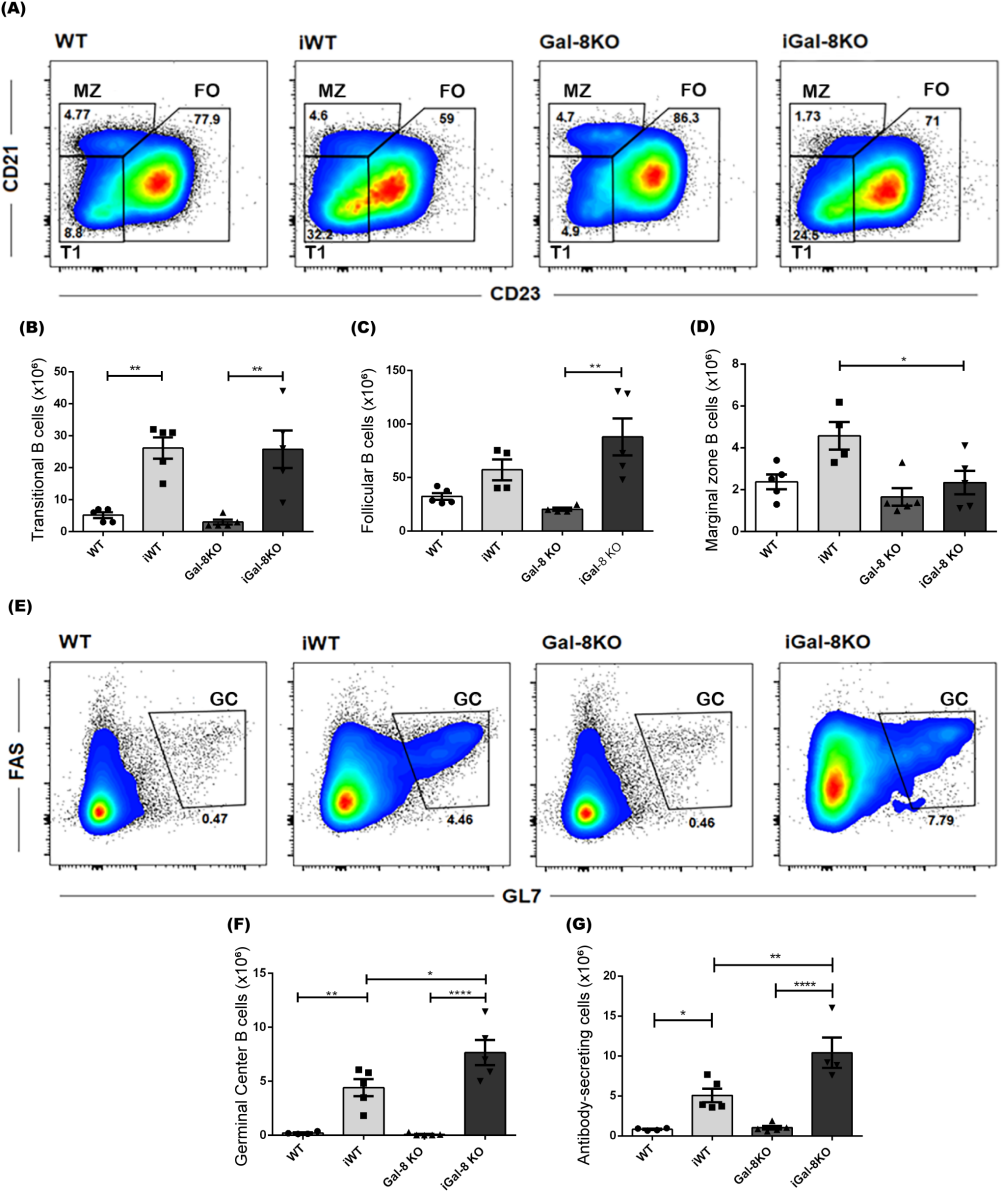
iGal-8KO mice present increased germinal center B cells and antibody secreting cell numbers. Evaluation, by flow cytometry, of different B cell subsets in the spleen of iWT and iGal-8KO mice obtained at 4 mpi. **(A)** Representative density dot plots of transitional B cells T1 (B220^+^ CD19^+^ CD21^low^ CD23^low^), FO (B220^+^ CD19^+^ CD21^int^ CD23^+^) B cells and MZ (B220^+^ CD19^+^ CD21^high^ CD23^low^) B cells. Statistical analysis of the absolute number of **(B)** T1 B cells/spleen, **(C)** FO B cells/spleen and **(D)** MZ B cells/spleen in infected mice and control groups. **(E)** Representative density dot plots of GC B cells (B220^+^ CD19^+^ FAS^+^ GL7^+^). Statistical analysis of the absolute number of **(F)** GC B cells/spleen and **(G)** ASC/spleen (B220^+^ CD138^+^). Data are expressed as mean ± SEM of a representative assay. At least three independent experiments were performed. Panels used for flow cytometry staining: CD21-FITC; B220-PE; CD23-PerCP; and CD19-PE-Cy7 **(A–D)**; CD19-FITC; FAS-PE; CD138-PerCP; B220-PE-Cy7; and GL7-APC **(E–G)**. Statistical significance of comparisons of mean values was assessed using one-way ANOVA followed by Bonferroni’s *post-hoc* test. *p<0.05; **p<0.01; ****p<0.0001.

Cell subsets were defined as follicular B cells (FO: B220^+^ CD19^+^ CD21^int^ CD23^+^), marginal zone B cells (MZ: B220^+^ CD19^+^ CD21^high^ CD23^low^), and transitional/immature B cells (T1: B220^+^ CD19^+^ CD21^low^ CD23^low^) (LeBien and Tedder, 2008) (**Figure 3A**). FO, the prevailing subset of the splenic B compartment, and T1 cells showed similar absolute numbers between infected mice (**Figures 3B, C**); whereas MZ cells were significantly decreased in iGal-8 KO mice compared to iWT (**Figure 3D**). The evaluation of the antibody-secreting cells (ASC) population, based on the expression of B220 and CD138 surface markers, together with the analysis of expressing B220^+^ FAS^+^ GL7^+^ germinal center B cells (GC), revealed that iGal-8KO mice had higher absolute numbers of ASC and GC in their spleen than iWT mice (**Figures 3E-G**). These data show that each infected group of mice presented consistent ASC and GC values.

**Figure 4:**
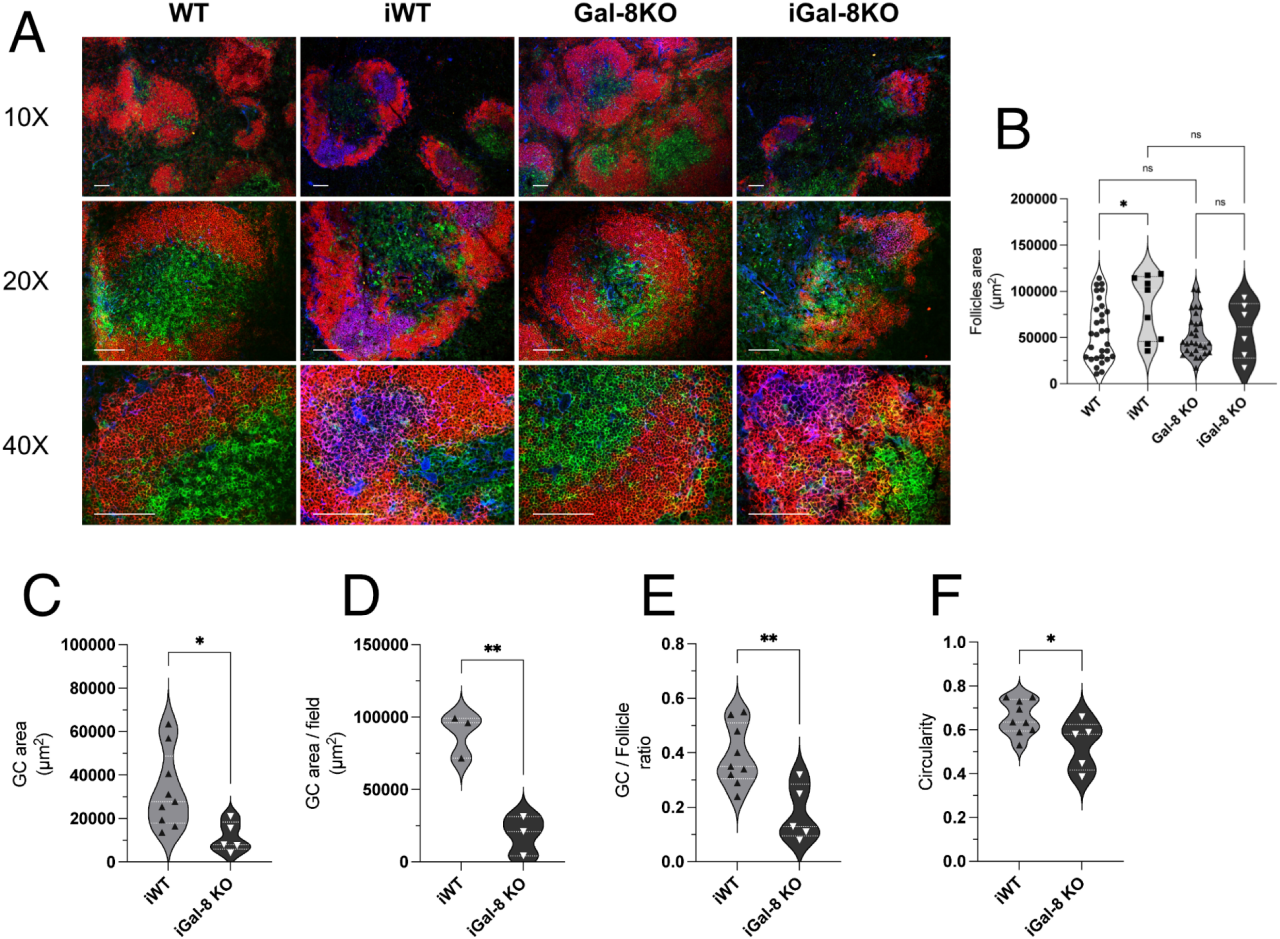
iGal-8KO mice present disorganized Germinal Centers in the chronic phase of the infection. **(A)** Immunofluorescence of spleen sections from iWT and iGal-8KO mice obtained at 4 mpi and their respective controls, stained with anti-B220 (red), anti-CD4 (green) and peanut agglutinin (PNA, blue). Note the presence of GC in the iWT and the disorganized GC in the iGal-8KO mice. Bar 100 µm. **(B)** Follicles area in infected and control mice groups. **(C)** GC area, **(D)** GC area per field, **(E)** GC-to-follicle area ratio in infected mice. **(F)** GC circularity in infected mice and control groups. Data from at least three independent animals per group were pooled for quantification. Data in **(B)** were analyzed using one-way ANOVA with Šídák’s multiple comparisons test. Data in all other graphs were analyzed using two-tailed Students’ *t*-test. *p<0.05; **p<0.01; ns, not significant.

Given the importance of architecture, polarization, and regionalization of GCs within B cell follicles in ensuring their functionality (Mesin et al., 2016; Cyster and Allen, 2019; Victora and Nussenzweig, 2022), we aimed to evaluate these aspects in iGal-8KO mice. To address this, we performed immunofluorescence staining on spleen sections from infected and uninfected mice (**Figure 4A**). The spleens of uninfected mice, both WT and Gal-8 KO, showed well-preserved follicular B cell structures with no evidence of GCs. As expected, spleens from iWT mice exhibited peanut agglutinin (PNA)-positive structures within follicles, forming GCs with a conserved, round architecture. In contrast, iGal-8KO mice displayed multiple heterogeneous PNA^+^ structures within B cell follicles, accompanied by marked microarchitectural disorganization. To quantitatively assess these differences, we measured several structural parameters. Follicle area did not differ significantly across groups (**Figure 4B**), while the total GC area itself (**Figure 4C**) and per field (**Figure 4D**) were significantly reduced in iGal-8KO mice compared to iWT controls. Furthermore, the ratio of GC to follicle area (**Figure 4E**), used to estimate the proportion of the follicle engaged in GC response, was markedly lower in iGal-8 KO mice. We also analyzed GC circularity (**Figure 4F**) as a geometric measure of GC organization and structural integrity. Well-formed GCs typically exhibit a round morphology, whereas irregular or fragmented GCs show reduced circularity. In line with this, GCs in iGal-8KO mice displayed significantly lower circularity values compared to iWT (**Figure 4F**). To study the functional capacity of GC we have analyzed the avidity of sera antibodies by ELISA and expressed them as the ratio of absorbance/absorbance after 6M urea treatment of sera (see Materials and Methods). Results obtained 1,871 ± 0,2071 (iWT) and 1,966 ± 0,1548 (iGal-8KO) showed no significative differences between infected groups (n=8 in each group).

#### 3.2.3 T lymphocytes as the main contributors to chronic splenomegaly persistence

**Figure 5:**
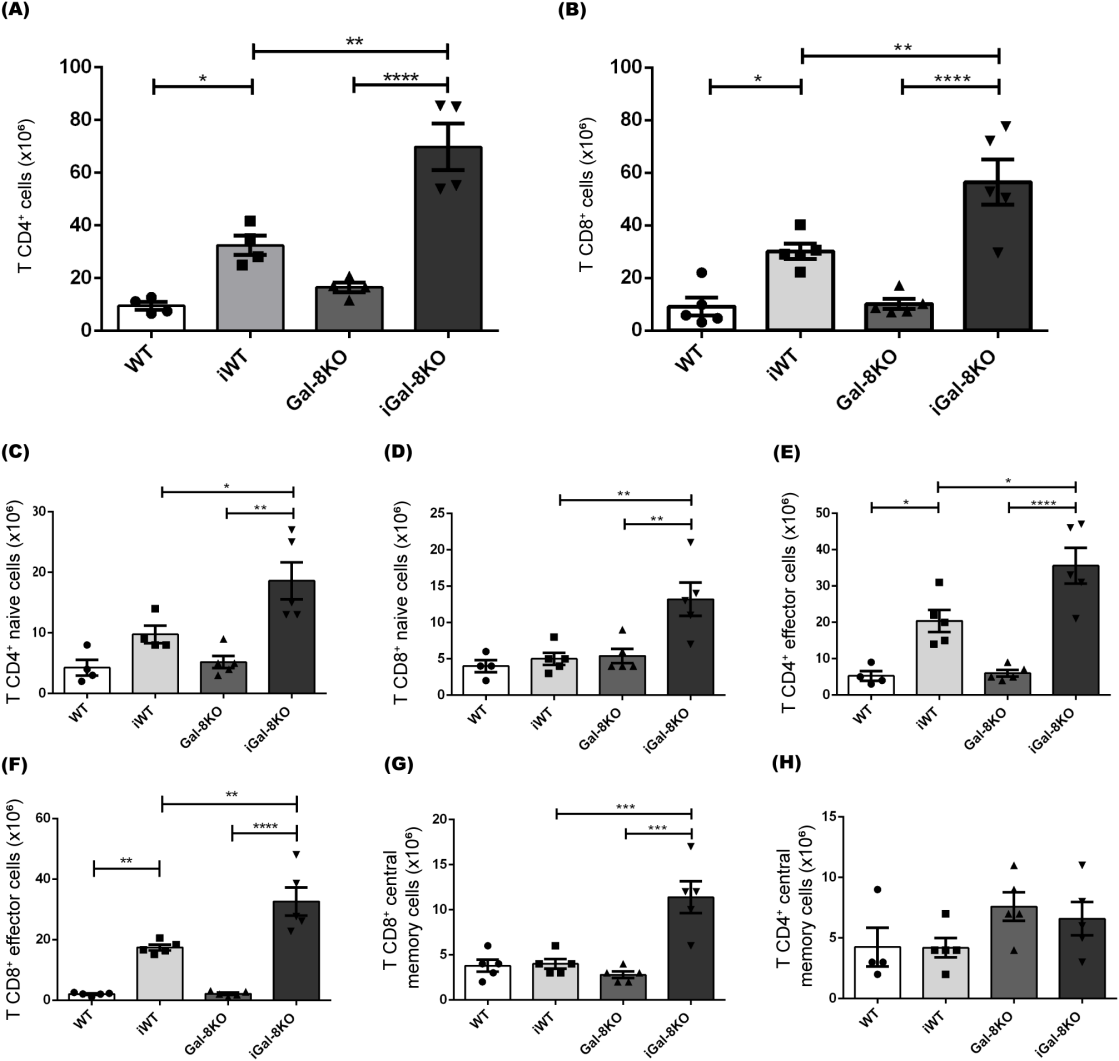
CD4^+^ and CD8^+^ T cells are significantly increased in iGal-8KO mice. The frequency of splenic T cell populations was analyzed by flow cytometry in iWT and iGal-8KO mice obtained at 4 mpi. Statistical analysis of the absolute number of **(A)** CD4^+^ T cells/spleen, **(B)** CD8^+^ T cells/spleen, **(C)** Naive CD4^+^ T cells/spleen (CD44^-^ CD62L^+^); **(D)** Naive CD8^+^ T cells/spleen (CD44^-^ CD62L^+^); **(E)** CD4^+^ effector T cells/spleen (CD44^+^ CD62L^-^); **(F)** CD8^+^ effector T cells/spleen (CD44^+^ CD62L^-^); **(G)** CD8^+^ central memory T cells/spleen (CD44^+^ CD62L^+^); **(H)** CD4^+^ central memory T cells/spleen (CD44^+^ CD62L^+^). Evaluation was carried out in the spleen of iWT and iGal-8KO mice 4 mpi. Data are expressed as mean ± SEM of a representative assay. At least three independent experiments were performed. Panel used for flow cytometry staining: CD44-FITC; CD62L-PerCP; CD8-APC; and CD4-APC-Cy7. Statistical significance of comparisons of mean values was assessed using one-way ANOVA followed by Bonferroni’s *post-hoc* test. *p<0.05; **p<0.01; ***p<0.001; ****p<0.0001.

Flow cytometry analysis revealed the expansion of CD4^+^ and CD8^+^ T cell populations after *T. cruzi* infection (**Figures 5A, B**). Notably, the absolute numbers of T cells were significantly higher in iGal-8KO mice compared to iWT.

T cells are classified into naïve, central memory and effector phenotypes based on the expression of CD62L (L-selectin) and CD44. Specifically, CD44^-^ CD62L^+^ cells are identified as naïve, CD44^+^ CD62L^+^ cells represent the central memory subset, and CD44^+^ CD62L^-^ cells correspond to the effector subpopulation (Nolz et al., 2011). The analysis of these subsets revealed that both CD4^+^ and CD8^+^ populations in iGal-8KO mice had significantly higher absolute numbers of naïve (CD44^-^ CD62L^+^) and effector (CD44^+^ CD62L^-^) T cells compared to iWT mice (**Figures 5C-F**). Only CD8^+^ central memory cells (CD44^+^ CD62L^+^) showed an increase in iGal-8KO mice (**Figure 5G**), whereas no differences were observed in CD4^+^ central memory cells between infected groups (**Figure 5H**).

**Figure 6:**
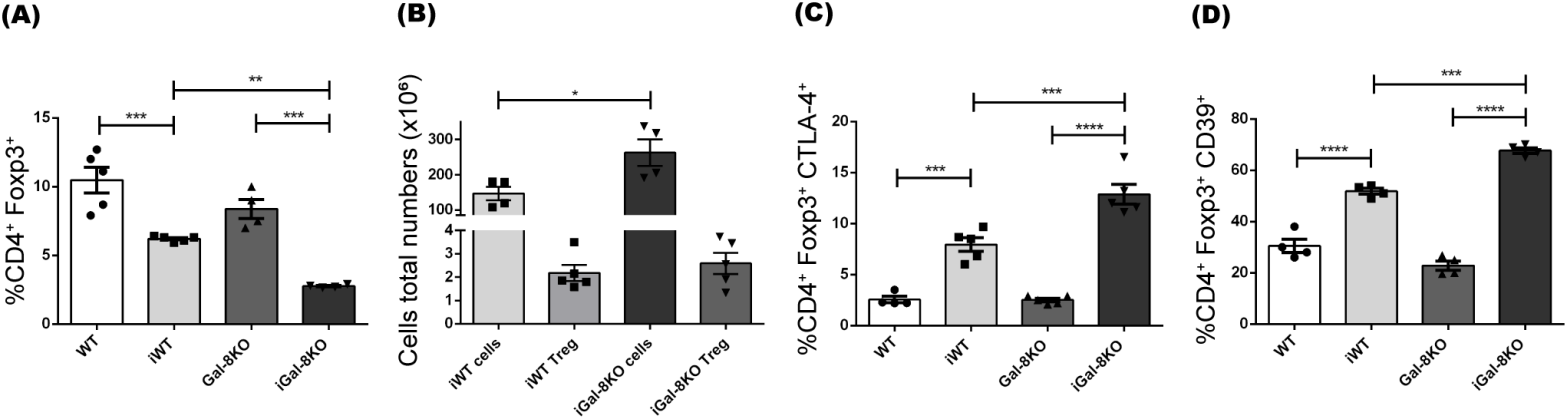
iGal-8KO mice did not show alterations in the development of regulatory T cells. **(A)** Frequency of regulatory T cells Foxp3^+^ (percentages depicted within CD4^+^ subset). **(B)** Absolute number of regulatory T cells/spleen (CD4^+^ Foxp3^+^) and its relationship with the total number of lymphoid cells/spleen, uninfected animals’ values were: WT cells: 58.91 ± 6.84; WT Treg: 1.24 ± 0.25; Gal-8KO cells: 49.4 ± 3.2; Gal-8KO Treg: 1.66 ± 0.25. **(C)** CTLA-4^+^ and **(D)** CD39^+^ expression in regulatory T cells (percentages depicted within CD4^+^ Foxp3^+^ subset). Evaluation was carried out in the spleens of iWT and iGal-8KO mice 4 mpi. Data are expressed as mean ± SEM of a representative assay. At least three independent experiments were performed. Panel used for flow cytometry staining: CTLA4-PE; FoxP3-PerCP; CD39-APC; and CD4-APC-Cy7. Statistical significance of comparisons of mean values was assessed using one-way ANOVA followed by Bonferroni’s *post-hoc* test **(A, C, D)** and two-tailed Student’s *t*-test **(B)**. *p<0.05; **p<0.01; ***p<0.001; ****p<0.0001.

It is known that a proper balance between Treg and effector T cells is important for controlling the magnitude and quality of the adaptative immune response. In our analysis, we observed a significant decrease in the frequency of total Foxp3^+^ Tregs in infected mice compared to their uninfected controls (**Figure 6A**). There were no differences in the absolute number of Tregs between iWT and iGal-8KO mice (**Figure 6B**). We then evaluated the expression of key functional suppressive proteins in Tregs, including CTLA-4 and CD39 (Takenaka et al., 2016). This revealed a significant increase in the percentage of Tregs expressing CTLA-4 and CD39 in iGal-8KO mice compared to iWT (**Figures 6C, D**).We next analyzed the CD4 T cell populations involved in supporting and regulating the humoral immune response. This analysis included T follicular helper cells (Tfh), which support GC responses and are characterized as CD4^+^ CXCR5^+^ ICOS^+^ Foxp3^-^, and T follicular regulatory cells (Tfr), which limit GC responses and are defined as CD4^+^ CXCR5^+^ ICOS^+^ Foxp3^+^ B220^-^ (Qi, 2016; Vinuesa et al., 2016). Significant differences were detected only between infected and uninfected mice (**Supplementary Figures 2A, B**). The coefficient between the frequency of Tfh and Tfr cells was evaluated (**Supplementary Figure 2C**), since it is a parameter associated with the amplitude of humoral immune responses (Xu et al., 2017; Fan et al., 2018). Despite this coefficient being increased in infected mice compared to their uninfected controls, no differences were observed between iWT and iGal-8KO mice (**Supplementary Figure 2C**). Results indicate that Gal-8 deficiency leads to an increase in effector T cell subsets without a change in Treg numbers, suggesting an imbalance in the T cell compartment.

**Figure 7:**
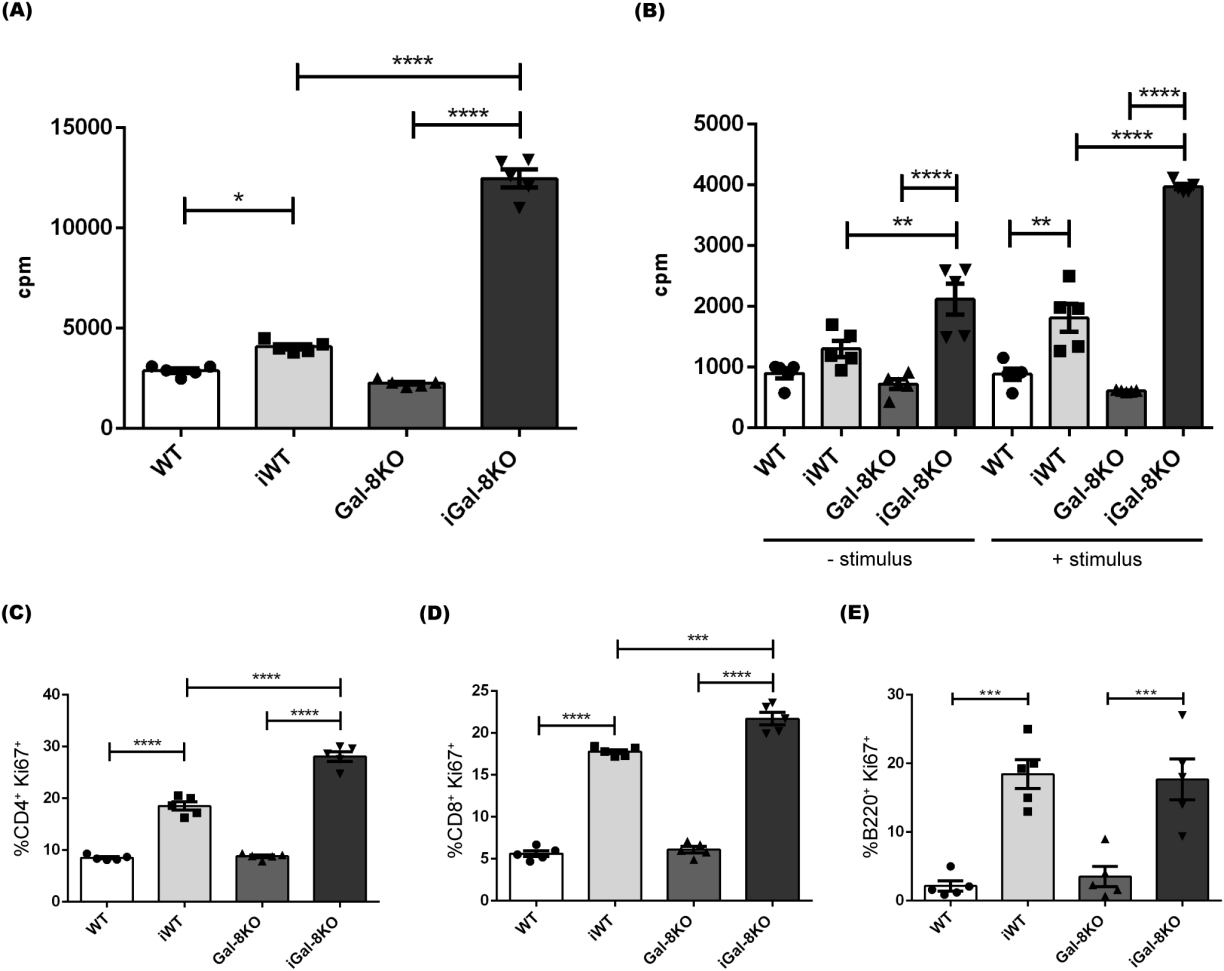
Splenocytes from iGal-8KO mice showed increased proliferation. Cells were obtained from spleens of iWT and iGal-8KO mice at 4 mpi and their respective control groups. Statistical analysis of **(A)** the basal proliferation index after 24 h of culture and **(B)** the basal proliferation index after 48 h of culture, with or without re-stimulation with specific antigen. Analysis was performed by incorporation of tritiated thymidine. Statistical analysis of the frequency of **(C)** Ki-67^+^ CD4^+^ T cells (percentages depicted within CD4^+^ subset), **(D)** Ki-67^+^ CD8^+^ T cells (percentages depicted within CD8^+^ subset) and **(E)** Ki-67^+^ B220^+^cells (percentages depicted within B220^+^ subset). Data are expressed as mean ± SEM of at least three independent experiments. Panel used for flow cytometry staining: Ki67-PE; CD8-APC; B220-PE-Cy7 and CD4-APC-Cy7. Statistical significance of comparisons of mean values was assessed using one-way ANOVA followed by Bonferroni’s *post-hoc* test. *p<0.05; **p<0.01; ***p<0.001; ****p<0.0001.

Next, the lymphocyte proliferative ability was analyzed in splenocyte cultures. Splenocytes obtained from infected and uninfected mice were allowed to proliferate for 24 (**Figure 7A**) and 48h (**Figure 7B**) without stimulus. When recently isolated, the iGal-8KO cells displayed a highly increased proliferative ability. Under stimulation with parasite antigens, iGal-8KO cells again showed significative increased proliferative activity respect to iWT cells. Several relevant cytokines (IL-2, IL-4, IL-6, IL-10, IL-17, TNF and IFNγ) levels were tested in the supernatants of splenocyte cultures stimulated with *T. cruzi* antigen (**Supplementary Figure 3**). Although all cytokine levels were increased in supernatants obtained from infected mice compared to their uninfected controls, only IL-6 showed higher levels in iGal-8KO supernatants compared to iWT.

To identify the proliferating cells, we analyzed the expression of Ki-67 marker in both B and T compartments (**Figure 7**). Flow cytometry analysis revealed a higher percentage of Ki-67^+^ CD4^+^ and Ki-67^+^ CD8^+^ T cells in the spleen of iGal-8KO mice in comparison to their counterpart in iWT (**Figures 7C, D**) consistent with the observed increase in the absolute number of these populations (**Figures 5A, B**). In contrast, B220^+^ proliferation rates were similar between infected mice (**Figure 7E**), which agrees with the previously described absolute numbers of B cells (**Supplementary Figure 1**). Thus, the differences in thymidine incorporation can be ascribed to T cell populations. Taken together, our results indicate that, beside the increased number of innate immune cells, CD4^+^ and CD8^+^ T cells emerge as the primary contributors to the persistence of chronic splenomegaly observed in iGal-8KO mice.

### 3.3 Gal-8 deficiency does not induce chronic splenomegaly in *Toxoplasma gondii* infection

To analyze whether the chronic persistence of splenomegaly was a widespread phenomenon that could be reproduced in the absence of Gal-8, we used another chronic infectious scenario, the infection with *Toxoplasma gondii*. This parasite induces a chronic murine infection characterized by acute splenomegaly, which begins to resolve at about 3 weeks post-infection (Li et al., 2021).

**Figure 8:**
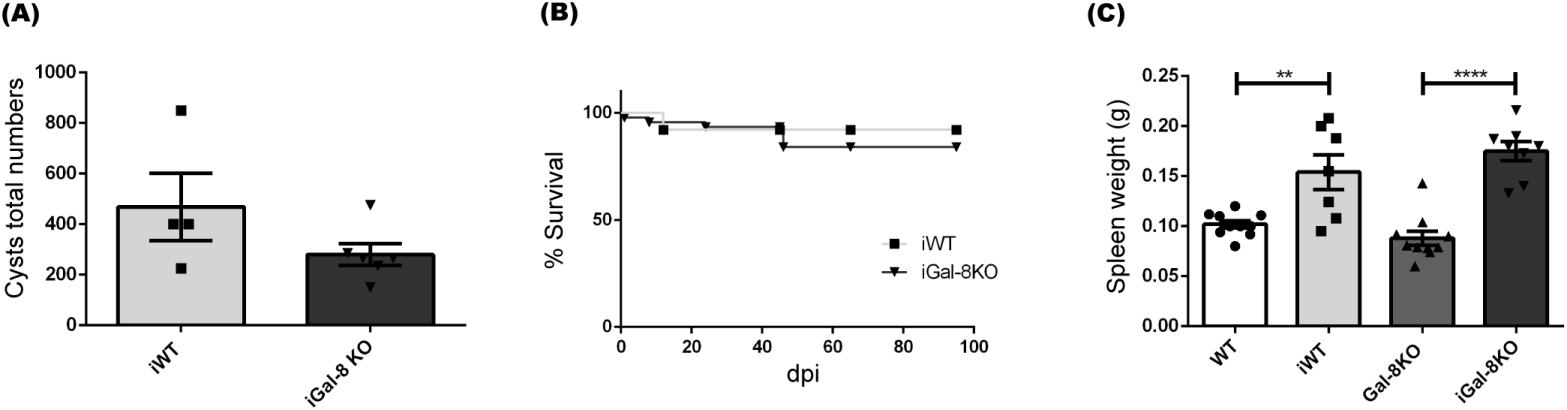
Gal-8 absence did not induce chronic splenomegaly during *Toxoplasma gondii* infection. WT and Gal-8KO mice were infected with the *T. gondii* Me49 strain and analysis was performed at 60 dpi. **(A)** Total numbers of cysts/brain. **(B)** Survival rate. **(C)** Spleen weight. Data are expressed as mean ± SEM of a representative assay. At least three independent experiments were performed. Statistical significance of comparisons of mean values was assessed using two-tailed Student’s *t*-test **(A)** and one-way ANOVA followed by Bonferroni’s *post-hoc* test **(C)**. Survival rates were compared using Gehan-Breslow-Wilcoxon test **(B)**. **p<0.01; ****p<0.0001.

The progression of *T. gondii* infection showed no variations between the infected groups. Parasite load, evaluated by counting brain cysts, revealed comparable values between iWT and iGal-8KO mice at 45, 60 and 90 dpi (**Figure 8**). Moreover, both infected groups displayed similar survival. Splenomegaly rates values were similar in both groups, over the 90 dpi tested (shown for 60 dpi in **Figure 8**). Flow cytometry analysis also revealed no significant differences in the absolute number of B cells, T cells (CD4^+^ and CD8^+^), monocytes, macrophages, neutrophils, and dendritic cells, (**Supplementary Figure 4**). These results show that the lack of Gal-8 is not a sufficient requirement to sustain chronic splenomegaly induced by *T. gondii*.

## 4 Discussion

The immune system is regulated by a complex network of interactors, including Gals. Currently, Gals are considered potential therapeutic targets to control chronic inflammatory processes in autoimmune or infectious diseases (Rabinovich and Toscano, 2009). In particular, the role of Gal-8 in the activation of the immune system is variable depending on the cellular context. While it promotes the activation of effector functions, such as inducing platelet activation (Romaniuk et al., 2010; Cattaneo et al., 2014), co-stimulation (Tribulatti et al., 2009) and T lymphocyte proliferation (Tribulatti et al., 2012), several studies have reported an immunosuppressive role for this galectin in Jurkat cells (Norambuena et al., 2009), in CD4^+^CD8^+^ thymocytes (Tribulatti et al., 2007) and in models of autoimmune diseases (Sampson et al., 2015; Pardo et al., 2017).

Our study on the role of Gal-8 in the inflammatory context induced during chronic *T. cruzi* infection showed its anti-inflammatory property (Bertelli et al., 2020). Here, we describe and analyze the persistence of splenomegaly in the chronic phase of *T. cruzi* infection in mice lacking Gal-8. Although there are several hypothesis and findings supporting the development of splenomegaly based in the high immune reactivity during the acute phase of the infection, there is scant information regarding the recovery of organ size. In our model, during the acute phase, the occurrence of splenomegaly, evaluated as organ weight, was similar between the infected groups (**Figure 1C**). After 2 mpi, and as it progresses towards the chronic phase, splenomegaly was reduced in iWT mice. However, in iGal-8KO mice this did not occurs (**Figures 1C-F**). At 4 mpi, we observed significant differences in the weight of the spleen and in the number of splenocytes in iGal-8KO animals compared to iWT (**Figures 1D-F**). This phenomenon is associated to the infection, since neither the weight nor the total number of splenocytes were altered by the absence of this galectin in uninfected mice. In human Chagas disease, splenomegaly has been described during the acute phase in infections acquired by different routes, including vectorial, congenital and organ transplants (Zaidenberg and Segovia, 1993; Moya et al., 2005; Bern et al., 2011; Cabrera et al., 2013; Fernandez-Villegas et al., 2014; Echeverria and Morillo, 2019). In all cases, splenomegaly resolves after the acute phase is controlled, as occurs in the mouse model. Therefore, this model could provide important clues to understanding the resolution of splenomegaly, the associated events of which are still unknown.

Lymphocytes B response to *T. cruzi* acquires an important role in controlling the spread and maintaining low levels of parasitemia in the chronic phase (Amezcua Vesely et al., 2012). At 4 mpi, the analysis of the expression of different phenotypic markers in B cells, allowed us to study the role of Gal-8 in the humoral immune response against the parasite. We observed an increased number of transitional B lymphocytes in infected mice (**Figure 3B**), that could be related with the known polyclonal activation of B cells (Amezcua Vesely et al., 2012). iGal-8KO mice showed reduced cell numbers in the MZ population (**Figure 3D**). It is possible that this population, that respond rapidly to stimuli circulating in the blood, quickly differentiate into ASC or GC B cells (Song and Cerny, 2003; Amezcua Vesely et al., 2012; Cerutti et al., 2013). Therefore, it is not surprising that a decrease in MZ B lymphocytes is associated with an increase in GC B cells. The observed reduction in the number of MZ B cells does not necessarily indicate that these cells have not exerted or are not exerting their function. Simultaneously, a significant increase in the number of mature B lymphocytes occurs, as consequence of cellular division (**Figures 3F, G**). Cytometry evaluation showed increased number of GC B lymphocytes and ASC in iGal-8KO *vs* iWT mice (**Figures 3F, G**). Surprisingly, the evaluation in serum of total IgGs level and IgG1, IgG2a, IgG2b specific anti-*T. cruzi* subclasses values were similar between iWT and iGal-8KO mice (data not shown) as well as sera antibodies avidity. However, immunohistochemical assays showed in iGal-8KO that GCs, identified as PNA^+^, were highly disorganized (**Figure 4**).

Gals have been involved in the development of B cells. Evidence have suggested that they play an important role in their signaling and activation (Acosta-Rodriguez et al., 2004; Beccaria et al., 2018) then they can influence cell fate decisions by regulating the balance between differentiation into memory or plasma cells. During *T. cruzi* infection, it was shown that Gal-3 in collaboration with IL-4, promotes differentiation into memory B cells at the expense of plasma cell differentiation (Acosta-Rodriguez et al., 2004). The absence of Gal-3 generates a higher number of ASC in response to other parasites challenge (Brand et al., 2012; Toscano et al., 2012) and also drives lupus-like disease by promoting spontaneous germinal centers in aged mice (Beccaria et al., 2018). In addition, Gal-1 and Gal-8 have been involved in the development of plasma cells. Gal-1 is significantly increased by B cell differentiation and is directly regulated by Blimp-1, in murine and human B cells (Tsai et al., 2008). However, Gal-1 would not be strictly essential since Gal-8 can functionally compensate for its absence, being the antibody production not affected (Tsai et al., 2011). In our model, we cannot rule out that the absence of Gal-8 might be compensated by another Gals. For instance, Gal-9, that belongs to the same family as Gal-8 and is related to the promotion, formation, differentiation, and survival of B-lineage cells, could be involved in these events (Cao et al., 2018). On the other hand, since Tfh cells are involved in GC formation, somatic hypermutation and affinity maturation (Qi, 2016), the significant increase of Tfh could impact promoting B-lineage development in iGal-8KO mice resulting in uncontrolled GC reactions (Sage and Sharpe, 2015).

The study of DCs showed their significant increase in the spleen of iGal-8KO mice (**Figure 3E**). These cells have the capacity to activate and become potent antigen presenters. DCs are known to secrete chemokines that recruit different cell populations of the innate response. In addition, Gals can inhibit or increase the activation of these cells (Tribulatti et al., 2020), which could explain the increase in the total number of monocytes and neutrophils in this organ (**Figures 2B, C**). In turn, Gals can also modulate key cellular activities that leads to leukocyte recruitment, probably by affecting chemoattraction and/or cell adhesion, potentially through both extracellular and intracellular signaling pathways (Liu and Stowell, 2023). Furthermore, the increase in these myeloid populations could be associated with a possible extramedullary myelopoiesis in the spleen (Swirski et al., 2009; Robbins et al., 2012; Zick, 2022). This event has been described in other infectious processes such as those triggered by *Plasmodium* sp., *Leishmania major* and *Ehrlichia muris* (Mirkovich et al., 1986; MacNamara et al., 2011; Belyaev et al., 2013). The absence of Gal-8 could be favoring this phenomenon. As mentioned before, the most striking immunological event of acute *T. cruzi* infection is the polyclonal activation of lymphocytes, leading to immunosuppression and splenomegaly (Minoprio et al., 1989). An intense proliferative activity of either B cells, CD4^+^ and CD8^+^ T cells, specific or nonspecific (Minoprio et al., 1986) is unleashed. As the infection progresses, a decrease in splenomegaly and a specific immune response is developed that is essential to control parasite replication and the survival of the host. The spleen reduction was observed in iWT mice from day 60 pi, however iGal-8KO mice failed to carry out it (**Figure 1C**). CD4^+^ and CD8^+^ T populations were increased in iGal-8KO mice compared to their iWT counterpart (**Figure 5**). Different authors have shown that Gal-8 is involved in Tregs differentiation modulating IL-2 and TGF-β signaling and thus, promoting cell death and inhibiting the proliferation of stimulated T cells through the increase of inhibitory molecules and the production of IL-10 (Sampson et al., 2016). This ability of Gal-8 generates in the murine model of autoimmune uveitis a decrease in the production of cytokines of the Th1 and Th17 pathways, favoring the decrease of the inflammatory process (Sampson et al., 2015). In addition, the role of Gal-8 as an immunosuppressor in experimental autoimmune encephalomyelitis was demonstrated. Mice deficient in this Gal show increased inflammatory levels leading to a more severe chronic phase due to a decrease in the regulatory population (Pardo et al., 2017). In addition, during *T. cruzi* infection, Tregs can induce a suppressive action on CD4^+^, CD8^+^ T lymphocytes and on other populations such as antigen-presenting cells (Araujo Furlan et al., 2018). In our *T. cruzi* chronic murine model Gal-8 showed anti-inflammatory properties (Bertelli et al., 2020), similar results were observed in a Gal-8 deficient murine model of autoimmunity (Pardo et al., 2017). However, we did not observe differences in the total numbers of regulatory T cells (**Figures 6A, B**) and IL-10 levels between infected groups (**Supplementary Figure 3**). When analyzing phenotypic characteristics related to functional status of these regulatory cells, we found an increase in CTLA-4 and CD39 (**Figures 6C, D**), which suggests that the overexpression of these molecules in iGal-8KO mice could be a compensatory effect, since the same number of regulatory cells are involved in controlling twice the number of cells (**Figure 6B**).

The study of splenocytes proliferation showed that levels were indeed significantly increased in iGal-8KO mice (**Figure 7**). Using Ki-67 we identified, by flow cytometry, that both CD4^+^ and CD8^+^ T-lineage populations were proliferating (**Figure 7**). The status of this lineage showed that effector and memory cells were increased in the spleens of iGal-8KO mice (**Figure 5**). Increased proliferation in Gal-8KO, was accompanied by an increase in the production of IL-6 (**Supplementary Figure 3**). IL-6 is a pleiotropic cytokine (Hunter and Jones, 2015) that enhances the immune response and has a strong pro-inflammatory ability (Gao and Pereira, 2002). While Gal-8 is known to exacerbate IL-6 production by DC stimulation (Carabelli et al., 2017; Carabelli et al., 2018), in this study we show that iGal-8KO mice produce higher levels of IL-6 than their iWT counterpart, supporting its induction by infection-derived stimuli. Depending on the context, IL-6 might play pro- or anti-inflammatory roles that can be protective in infectious diseases it helps solve, but deleterious in autoimmune affections associated with the chronic presence of this cytokine (Hunter and Jones, 2015). IL-6 is crucial for host survival from *T. cruzi* infection, as its absence leads to increased parasitemia and earlier mortality, appearing to play a role as a direct mediator of inflammation (Gao and Pereira, 2002). In our study we observed sustained IL-6 presence in the chronic phase of infection. Considering the persistent splenomegaly in the absence of Gal-8 and the increased titter of IL-6, it appears that a chronic inflammatory effect can also be attributed to this cytokine. Additionally, Gal 8 seems to modulate IL 6 production. Thus, IL-6 appears to be involved in both stages of infection, playing a protective role during the acute phase, and a detrimental one in the chronic phase, similar to what occurs during its sustained presence in several autoimmune diseases.

Gal-8 has been reported to induce apoptosis via different pathways such as the accumulation of p21, a cyclin-dependent kinase inhibitor that exhibits strong anti-apoptotic activity (Hadari et al., 2000; Arbel-Goren et al., 2005). CD4^high^CD8^high^ thymocytes enter apoptosis via caspases activation, after being stimulated with Gal-8 (Tribulatti et al., 2020). Gal-8 stimulation in Jurkat T cells induces apoptosis via the phosphatidic acid mediated ERK1/2 activation pathway (Norambuena et al., 2009). It is interesting to highlight that different groups have demonstrated that human peripheral blood mononuclear cells (PBMCs) must be activated either by PHA or anti-CD3/CD28, in order to be sensitive to apoptosis induced by Gal-8 (Norambuena et al., 2009; Cattaneo et al., 2011). Then, it was proposed that Gal-8, together with other galectins that are able to kill activated T cells, contribute to T cells homeostasis (Norambuena et al., 2009). Given that the persistent increased cellularity in the spleens from iGal-8KO mice corresponds mainly to the T cell population (**Figure 5**), we could hypothesize that this excessive proliferation would be related to the inability to activate apoptosis pathways.

In our efforts to deepen our knowledge of the role of this galectin in the development of chronic splenomegaly, we extended our analysis to chronic *T. gondii* infection in Gal-8KO and the counterpart mice. The spleen weight and cytometry populations studies didn’t show differences between infected groups at different times of infection (**Supplementary Figure 4**), indicating that the induced splenomegaly persistence in Gal-8KO mouse is not a general event but a result of the interaction with the *T. cruzi* infection process.

Another issue to take in consideration, is that the absence of Gal-8 didn’t modify parasitemia values nor mortality rate in *T. cruzi*-infected mice (Bertelli et al., 2020). Likewise, similar number of cerebral cysts and mortality values were observed in *T. gondii*-infected mice. Taken together, we can speculate that Gal-8 is not related to the events involved in protection, in the analyzed models. A compensatory mechanism by another galectin cannot be ruled out.

The Gal-8KO mouse model provides evidence supporting that the retraction of the acute splenomegaly is strongly related to Gal-8 participation during the *T. cruzi* infection, a parasite known as a stronger inducer of inflammation.

## 5 Conclusions

-The murine model used allowed us, for the first time, to associate a specific molecule, Gal-8, with the resolution of acute splenomegaly induced by *Trypanosoma cruzi* infection.

-The absence of Gal-8 did not modify parasitic load or mortality rate in either *Trypanosoma cruzi*- or *Toxoplasma gondii*-infected mice.

-The generation of germinal centers is hampered in the absence of Gal-8 during the infection with *Trypanosoma cruzi.*

-The absence of Gal-8 promotes increased proliferation of CD4^+^ and CD8^+^ T cells during *Trypanosoma cruzi* infection.

-Gal-8 plays an anti-inflammatory role in *Trypanosoma cruzi* infection.

## Data Availability

The original contributions presented in the study are included in the article/**Supplementary Material**. Further inquiries can be directed to the corresponding author.
