# Supplementary material for: Galectin-8 deficiency promotes chronic splenomegaly persistence in Chagas disease
Source: Front Cell Infect Microbiol. 2025 Oct 1;15:1625938. doi: 10.3389/fcimb.2025.1625938 (PMC12521124; doi:10.3389/fcimb.2025.1625938)
Supplement: Supplementary file 1 [file DataSheet1.pdf]

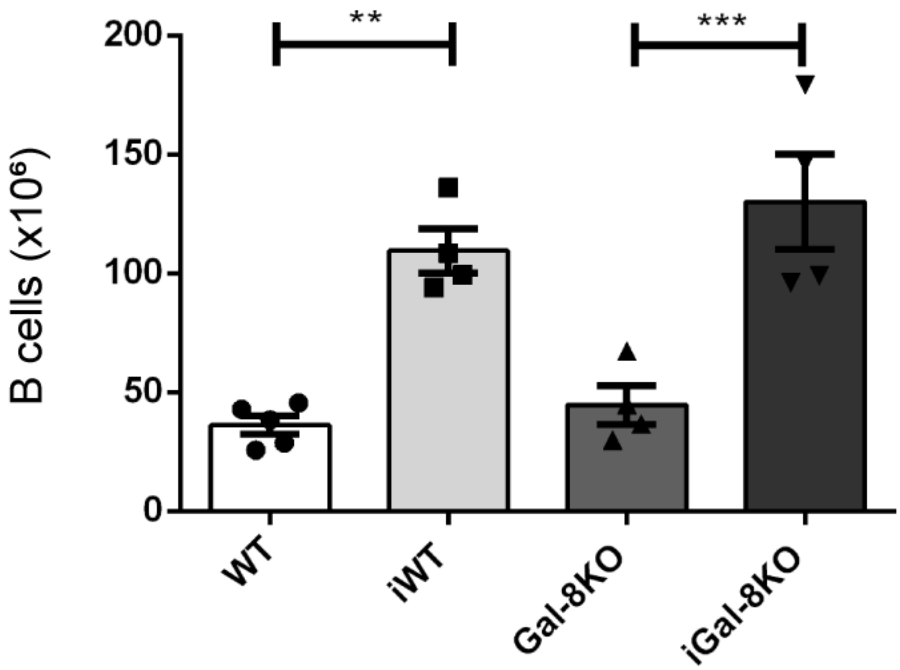

Supplementary Figure 1: **iGal-8KO and iWT mice exhibit similar numbers of B220⁺ cells**. Statistical analysis of the absolute number of B cells (B220⁺). The evaluation was carried out in the spleen of iWT and iGal-8 KO mice 4 mpi and their respective control groups. Data are expressed as mean ± SEM of at least three independent experiments. ***p*<0.01; ****p*<0.001.
