# Supplementary material for: Galectin-8 deficiency promotes chronic splenomegaly persistence in Chagas disease
Source: Front Cell Infect Microbiol. 2025 Oct 1;15:1625938. doi: 10.3389/fcimb.2025.1625938 (PMC12521124; doi:10.3389/fcimb.2025.1625938)
Supplement: Supplementary file 2 [file DataSheet2.pdf]

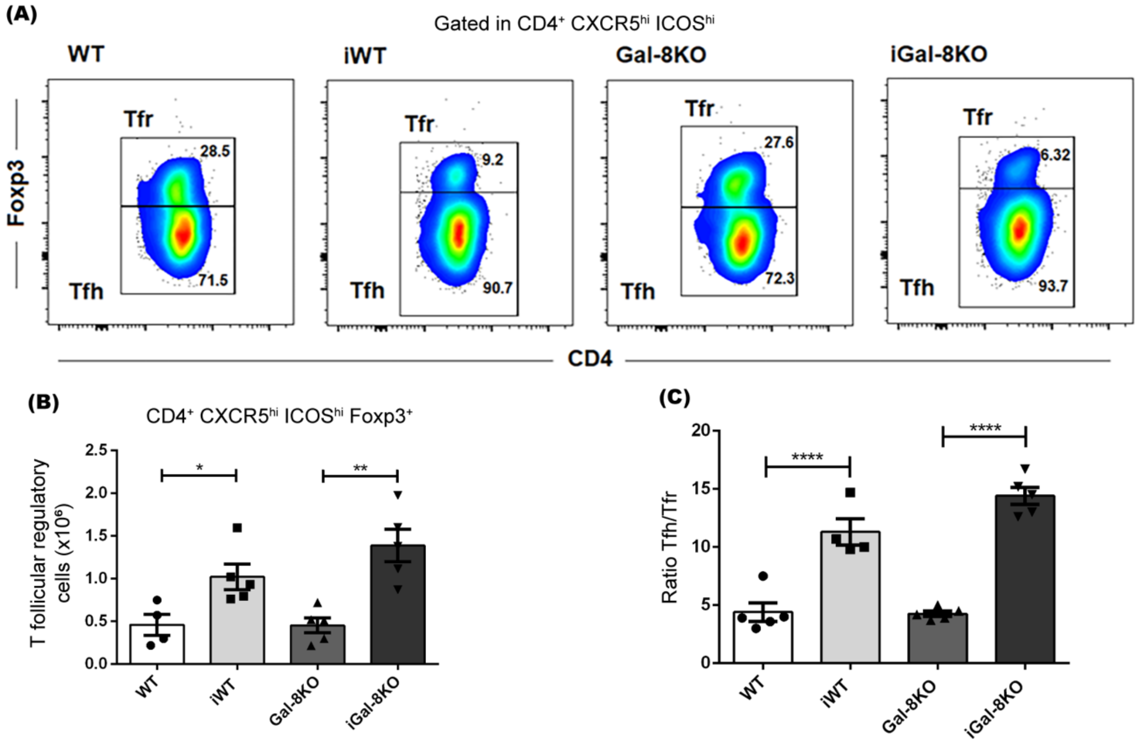

Supplementary Figure 2: iGal-8KO mice did not present alterations in the control of the magnitude of the humoral response.

The evaluation was carried out in the spleen of iWT and iGal-8 KO mice 4 mpi and their respective control groups. (A) Representative density dot plots of Foxp3^{+} *vs* CD4^{+} cells, depicted within CD4^{+} CXCR5^{hi} ICOS^{hi} subset. Statistical analysis of (B) T Follicular regulatory cells (Tfr)/spleen (CD4^{+} CXCR5^{hi} ICOS^{hi} Foxp3^{+}) and (C) the Tfh/Tfr cell ratio in the spleen of iWT, iGal-8KO, WT and Gal-8KO. Panel used for flow cytometry staining: FoxP3-PerCP; ICOS-PE*Cy7; CXCR5-APC; and CD4-APC*Cy7. Statistical significance of comparisons of mean values was assessed using one-way ANOVA followed by Bonferroni's *post-hoc* test. *$p<0.05$; **$p<0.01$; ****$p<0.0001$.
