# Supplementary material for: Galectin-8 deficiency promotes chronic splenomegaly persistence in Chagas disease
Source: Front Cell Infect Microbiol. 2025 Oct 1;15:1625938. doi: 10.3389/fcimb.2025.1625938 (PMC12521124; doi:10.3389/fcimb.2025.1625938)
Supplement: Supplementary file 3 [file DataSheet3.pdf]

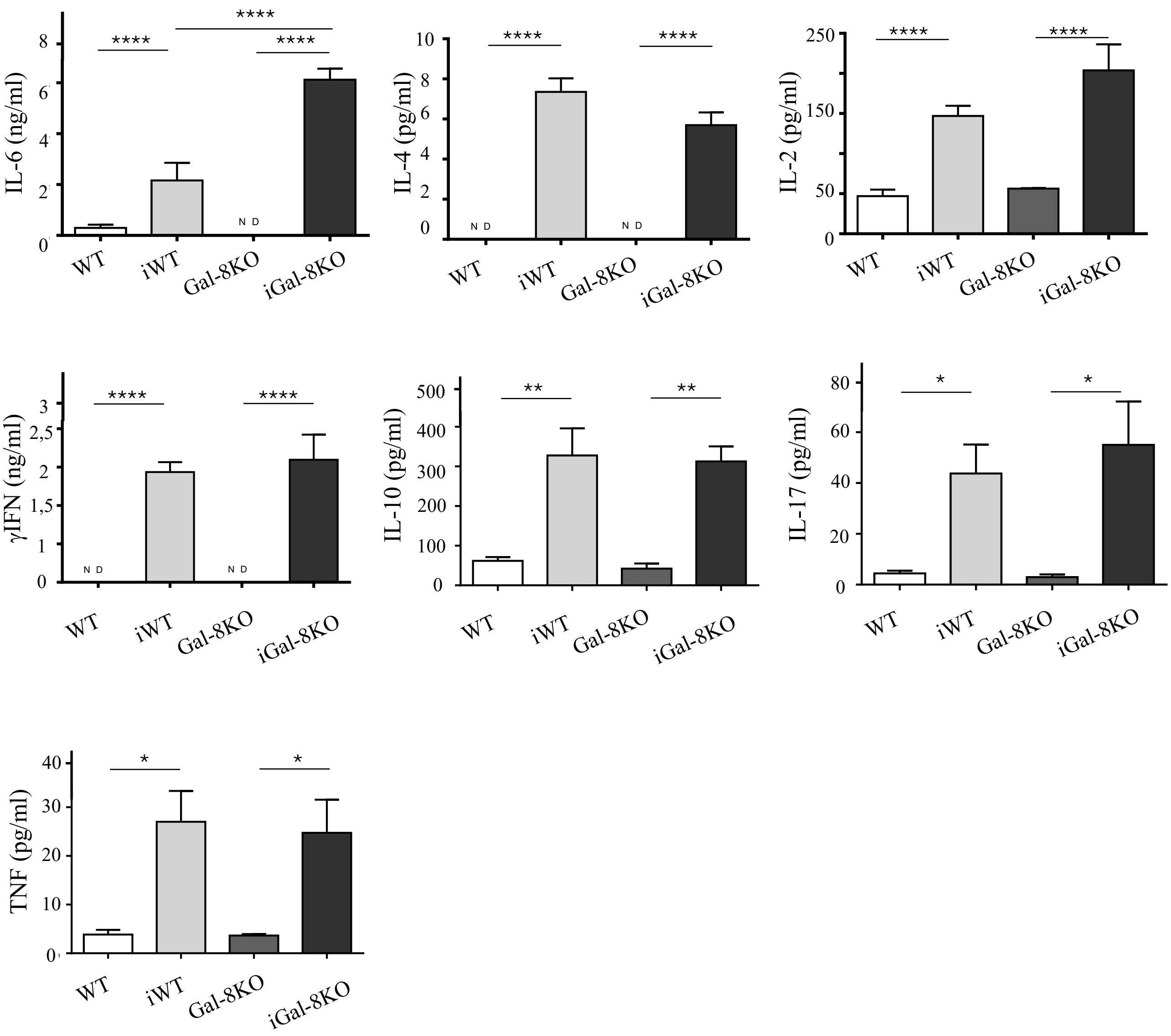

Supplementary Figure 3: Secreted cytokines values from *Trypanosoma cruzi*-infected mice. Splenocytes were obtained from infected mice at 4 mpi and control animals. Cells were cultured with *T. cruzi* stimulation and supernatants collected 48h later and assayed for the indicated interleukins by ELISA. Note that only IL-6 concentration was found significantly different between iWT and iGal-8KO cell cultures. ND: not detected. *p<0.05; **p<0.01; ****p<0.0001.
