# Supplementary material for: Galectin-8 deficiency promotes chronic splenomegaly persistence in Chagas disease
Source: Front Cell Infect Microbiol. 2025 Oct 1;15:1625938. doi: 10.3389/fcimb.2025.1625938 (PMC12521124; doi:10.3389/fcimb.2025.1625938)
Supplement: Supplementary file 4 [file DataSheet4.pdf]

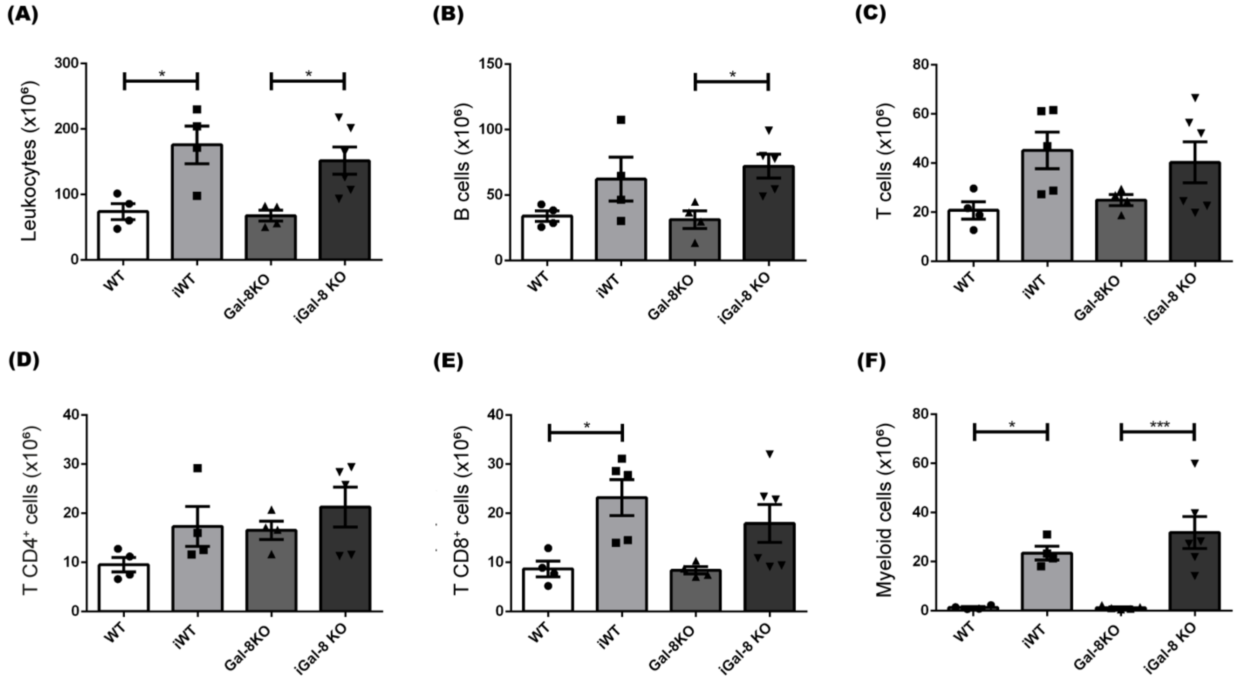

Supplementary Figure 4: Splenocyte populations collected from *Toxoplasma gondii*-infected mice. Different leukocyte subpopulations were evaluated at 90 dpi in animals naïve or infected with *T. gondii*. *p<0.05, ***p<0.001. No significant differences were observed between iGal-8KO *vs.* iWT mice (ANOVA).
